# Severe Scoliosis As the Clue for an Early Onset Myopathy, Areflexia, Respiratory Distress, and Dysphagia (EMARDD) Diagnosis During Childhood: A Case Report

**DOI:** 10.7759/cureus.74966

**Published:** 2024-12-02

**Authors:** Ana Sofia Figueiredo, Juliana Da Silva Cardoso, Manuela Santos, Cristina Garrido

**Affiliations:** 1 Pediatrics, Hospital de São Pedro, Unidade Local de Saúde de Trás-os-Montes e Alto Douro, Vila Real, PRT; 2 Paediatric Neurology, Centro Materno Infantil do Norte, Unidade Local de Saúde de Santo António, Porto, PRT

**Keywords:** areflexia, congenital myopathy, dysphagia, pediatric scoliosis, respiratory insufficiency

## Abstract

We present a novel case of a school-aged boy with scoliosis and progressive muscle weakness, featuring new onset hypotonia and respiratory distress. Genetic analysis revealed two heterozygous variants in the MEGF10 gene: one known pathogenic variant and one novel missense variant. This case illustrates the heterogeneous phenotype of early onset myopathy, areflexia, respiratory distress, and dysphagia (EMARDD), according to the mutations associated, and underscores the importance of early genetic testing. Among the few cases described in the literature, few report symptom onset and diagnoses after the first years of life, unlike the case reported here. Additionally, this report alerts for the suspicion of myopathy in children with severe scoliosis and recurrent respiratory infections and revises the current knowledge of EMARDD, emphasizing the necessity for comprehensive and timely treatment approaches.

## Introduction

Early onset myopathy, areflexia, respiratory distress, and dysphagia (EMARDD) is a rare congenital myopathy caused by mutations in the MEGF10 gene on chromosome 5q23 [1]. It represents an autosomal recessive disease associated with homozygous or compound heterozygous mutations [2]. EMARDD has recently been classified as a primary satellite cellopathy, integrating a group of congenital myopathies characterized by generalized muscle weakness and specific impairment of the respiratory, trunk, and facial muscles, typically with normal or slightly elevated serum creatine kinase (CK) levels [3].

The exact prevalence of EMARDD is not well established, but less than 100 patients have been documented in the literature to date [4,5].

The MEGF10 gene encodes a transmembrane protein highly expressed in skeletal muscle, playing a crucial role in satellite cell proliferation and migration during muscle regeneration. The loss of MEGF10 function is thought to affect the initial stages of muscle repair [4,6,7].

The first description of EMARDD in 2007 described patients with profound childhood weakness, hypotonia, and respiratory distress due to diaphragmatic paralysis, often requiring ventilatory support within the first months or years of life and never achieving ambulation [8]. This severe variant described was associated with homozygous or compound heterozygous mutations in the MEGF10 gene [6]. A few years later, a milder chronic variant (mvEMARDD) was identified, with a later onset and a relatively more favorable disease course. mvEMARDD patients often exhibit mild muscle weakness and moderate respiratory issues [8,9]. Concerning genotype-phenotype correlation, researchers found that individuals with missense mutations in at least one allele tended to have a significantly later onset than those with biallelic truncation mutations [8]. This finding suggests a potential link between the type of genetic variant, age at symptom onset, and disease severity in EMARDD [8].

In the context of neuromuscular diseases, scoliosis arises from a combination of factors such as diminished muscle strength and tone, impaired sensory feedback, and abnormal spinal balance. Together, these elements can lead to significant spinal asymmetry as the body grows [6,8,10]. Specifically, in EMARDD syndrome, evidence points to altered muscle strength and tone as the main factors contributing to scoliosis [6,8,10]. The muscular weakness in EMARDD affects the neck, arms, lower limbs, and diaphragm, which can also result in respiratory distress [4,8].

A recent comprehensive review by Allam and Schwabe (2013) documented 31 cases of MEGF10 myopathy reported across various studies [10]. The majority of pediatric patients described to date presented with symptoms within the first two years of life.

We present a case of a 9-year-old boy who was diagnosed later in childhood with severe scoliosis.

## Case presentation

The patient’s clinical journey began with a fetal intrauterine growth restriction diagnosed in the last 2 weeks of gestation and delivery at 37 weeks of gestation, born to nonconsanguineous parents. The neonatal period was uncomplicated, with no oxygen requirement or ventilatory support, discharged 3 days after birth.

During the first months of life, he was diagnosed with mild hypotonia and congenital torticollis that resolved after physiotherapy treatment.

During his first two years, the patient experienced several mild respiratory infections that did not require hospitalization. At the age of 4, he was diagnosed with mild scoliosis. At the beginning of elementary school, teachers reported major learning difficulties.

**Figure 1:**
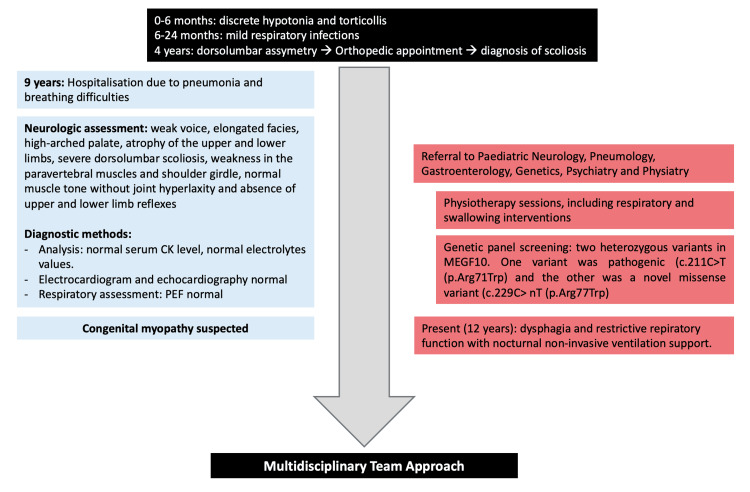
Case report timeline. Case report timeline describing symptoms and investigation upon hospitalization (CAse Report (CARE) Guidelines). PEF: peak expiration flow; CK: creatine kinase

At age 9, he was hospitalized due to pneumonia associated with respiratory distress. The presence of moderate scoliosis, muscular hypotrophy, and respiratory distress led to the suspicion of a neuromuscular disease. He was observed by neurology. At the physical exam, his height and weight were below the 10th percentile according to World Health Organization standards [11]. The initial neurological assessment revealed several significant findings: a weak voice, elongated facies, high-arched palate, atrophy of the upper and lower limbs, severe thoracolumbar scoliosis, weakness in the paravertebral muscles and shoulder girdle, normal muscle tone without joint hyperlaxity, and absence of upper and lower limb reflexes. The patient denied experiencing dysphagia or breathing difficulties (Figure 1).

**Figure 2:**
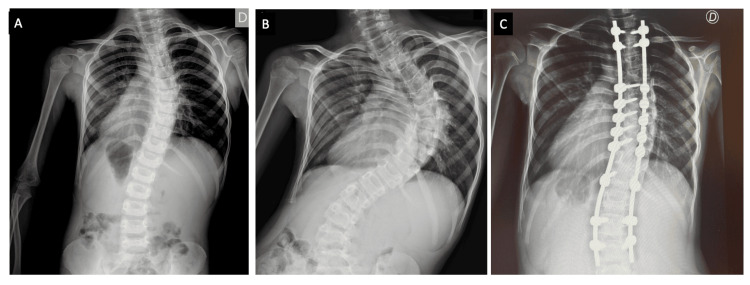
Patient scoliosis radiographs demonstrate the progression of scoliosis over time. A) Scoliosis at 5 years of age. B) Severe thoracolumbar scoliosis at 10 years of age, with a Cobb angle of 45 degrees. C) Post-surgical radiograph showing correction of the deformity.

First-line investigation revealed a normal serum CK level, normal cardiac evaluation (including electrocardiogram and echocardiography), and a respiratory assessment showing maximum instantaneous expiratory flow within normal limits. The scoliosis progressively worsened and was surgically interventioned by the age of 10 (Figure 2).

Following discharge, genetic testing was pursued to confirm the diagnosis. A genetic panel screening for hereditary myopathies, consisting of 154 genes, revealed two heterozygous variants in MEGF10. One variant was identified as pathogenic (c. 211C>T (p.Arg71Trp)), previously associated with a patient presenting a mild variant of the disease. The other mutation was a novel missense variant (c.229C>T (p.Arg77Trp)), which is currently classified as a variant of uncertain significance [12].

At 12 years of age, one year after genetic results, he developed dysphagia and restrictive respiratory function, requiring noninvasive nocturnal ventilation support. At present, therapeutic interventions include intensive physiotherapy, encompassing swallowing exercises to improve the coordination and strength of the tongue, lips, and throat muscles; postural adjustments to reduce the risk of aspiration and enhance swallowing efficiency; dietary texture modifications and compensatory techniques such as alternating food with liquid and employing multiple swallows per bite. Psychological support was very important in the early stages after diagnosis, assisting patients and parents in coping with symptoms and the progression of the disease.

## Discussion

This case reflects a rare inherited neuromuscular disorder that typically manifests in early childhood and affects muscle structure and function [4,13]. In the context of EMARDD, typical clinical presentations include muscle hypotonia and respiratory difficulties within the first few months of life. However, our patient exhibited an atypical onset, presenting with scoliosis without limb weakness at the age of 4 years.

Muscle weakness encompasses a broader spectrum of differential diagnoses, including inflammatory myopathies and numerous neuromuscular diseases. Scoliosis can develop in congenital myopathies and tends to appear especially early in the more severe ryanodine receptor-1-related myopathy and nemaline myopathy [14,15].

Nevertheless, there are no known specific ages for scoliosis to manifest in EMARDD syndrome; we understand that depending on the severity of muscle weakness, it can be a later presentation.

Serum CK is an important marker to evaluate, as it is normal or mildly elevated in congenital myopathies but raised in muscular dystrophy (usually more than five times the reference value). Traditionally, muscle biopsy has been pivotal in the diagnosis of congenital myopathies. Nevertheless, this patient did not perform a muscle biopsy since EMARDD muscle biopsies often exhibit nonspecific damage, according to several reports. These nonspecific findings highlight the rising need for genetic examinations in suspected cases [13,15].

The management of EMARDD requires a comprehensive multidisciplinary approach, including: Neurology: assessing muscle involvement and disease progression; Pulmonology: Monitoring respiratory function, managing ventilatory support, and preventing infections; Gastroenterology: Evaluating dysphagia and considering feeding tube placement; Physiatry: Developing muscle strengthening plans and swallowing training; Orthopedics: Treating and monitoring scoliosis, with potential surgical intervention; Psychiatry and Psychology: Helping patients manage expectations and cope with the disease course.

## Conclusions

This case highlights the late presentation of a rare neuromuscular disorder, with subtle symptoms since early childhood that were misdiagnosed, and reinforces the need to closely follow up with patients with recurrent lung infections and scoliosis. It is important to note that other conditions can present with similar symptoms, so a thorough medical evaluation is often necessary in the suspected cases. Since there are no targeted therapies, treatment primarily focuses on supportive measures, highlighting the importance of multidisciplinary care and interventions. Ongoing research into MEGF10 mutations holds promise for identifying potential therapeutic targets in the future.

## References

[1] Chikkannaiah M, Reyes I (2021). New diagnostic and therapeutic modalities in neuromuscular disorders in children. Curr Probl Pediatr Adolesc Health Care.

[2] (2024). OMIM. https://omim.org/entry/620249.

[3] Ganassi M, Muntoni F, Zammit PS (2022). Defining and identifying satellite cell-opathies within muscular dystrophies and myopathies. Exp Cell Res.

[4] AlMuhaizea M, Dabbagh O, AlQudairy H (2021). Phenotypic variability of MEGF10 variants causing congenital myopathy: Report of two unrelated patients from a highly consanguineous population. Genes.

[5] Lin YF, Wu XY, Yang L, Cheng GQ, Huang Y, Zhuang DY (2023). A family with early onset myopathy caused by MEGF10 gene defect and literature review. (Article in Chinese). Zhonghua Er Ke Za Zhi.

[6] Logan CV, Lucke B, Pottinger C (2011). Mutations in MEGF10, a regulator of satellite cell myogenesis, cause early onset myopathy, areflexia, respiratory distress and dysphagia (EMARDD). Nat Genet.

[7] (2024). ClinVar. https://www.ncbi.nlm.nih.gov/clinvar/RCV000023958/.

[8] Fujii K, Hirano M, Terayama A, Inada R, Saito Y, Nishino I, Nagai Y (2022). Identification of a novel mutation and genotype-phenotype relationship in MEGF10 myopathy. Neuromuscul Disord.

[9] Takayama K, Mitsuhashi S, Shin JY (2016). Japanese multiple epidermal growth factor 10 (MEGF10) myopathy with novel mutations: A phenotype-genotype correlation. Neuromuscul Disord.

[10] Allam AM, Schwabe AL (2013). Neuromuscular scoliosis. PM R.

[11] (2024). World Health Organization. https://www.who.int/tools/growth-reference-data-for-5to19-years/indicators/height-for-age.

[12] Colombo I, Scoto M, Manzur AY (2015). Congenital myopathies: Natural history of a large pediatric cohort. Neurology.

[13] Rolton D, Nnadi C, Fairbank J (2014). Scoliosis: A review. Paediatrics and Child Health.

[14] North KN, Wang CH, Clarke N (2014). Approach to the diagnosis of congenital myopathies. Neuromuscul Disord.

[15] Croci C, Traverso M, Baratto S (2022). Congenital myopathy associated with a novel mutation in MEGF10 gene, myofibrillar alteration and progressive course. Acta Myol.

