# Correction: Severe Scoliosis As the Clue for an Early Onset Myopathy, Areflexia, Respiratory Distress, and Dysphagia (EMARDD) Diagnosis During Childhood: A Case Report

**DOI:** 10.7759/cureus.c202

**Published:** 2024-12-09

**Authors:** Ana Sofia Figueiredo, Juliana Da Silva Cardoso, Manuela Santos, Cristina Garrido

**Affiliations:** 1 Pediatrics, Hospital de São Pedro, Unidade Local de Saúde de Trás-os-Montes e Alto Douro, Vila Real, PRT; 2 Paediatric Neurology, Centro Materno Infantil do Norte, Unidade Local de Saúde de Santo António, Porto, PRT

This article has been corrected to replace Figure 2 as the originally-included image did not show the post-surgical correction as stated. Figure 2, along with the corresponding figure title and legend, has now been updated to ensure accuracy.

